# Suppression of Hyperpolarization-Activated Cyclic Nucleotide-Gated Channel Function in Thalamocortical Neurons Prevents Genetically Determined and Pharmacologically Induced Absence Seizures

**DOI:** 10.1523/JNEUROSCI.0896-17.2018

**Published:** 2018-07-25

**Authors:** François David, Nihan Çarçak, Szabina Furdan, Filiz Onat, Timothy Gould, Ádám Mészáros, Giuseppe Di Giovanni, Vivian M. Hernández, C. Savio Chan, Magor L. Lőrincz, Vincenzo Crunelli

**Affiliations:** ^1^Neuroscience Division, School of Biosciences, Cardiff University, Cardiff CF10 3AX, United Kingdom,; ^2^Lyon Neuroscience Research Center, CNRS UMR 5292-INSERM U1028-Université Claude Bernard, 69008 Lyon, France,; ^3^Department of Pharmacology, Faculty of Pharmacy, Istanbul University, Istanbul, Turkey,; ^4^Department of Physiology, Anatomy, and Neuroscience, University of Szeged, Szeged 6726, Hungary,; ^5^Department of Pharmacology and Clinical 34452 Pharmacology, Marmara University School of Medicine, Istanbul 81326, Turkey,; ^6^Department of Physiology and Biochemistry, University of Malta, Msida MSD 2080, Malta, and; ^7^Department of Physiology, Feinberg School of Medicine, Northwestern University, Robert H Lurie Medical Research Center, Chicago, Illinois 60611

**Keywords:** absence epilepsy, channelopathy, HCN channels, thalamocortical rhythms, thalamus

## Abstract

Hyperpolarization-activated cyclic nucleotide-gated (HCN) channels and the *I*_h_ current they generate contribute to the pathophysiological mechanisms of absence seizures (ASs), but their precise role in neocortical and thalamic neuronal populations, the main components of the network underlying AS generation, remains controversial. In diverse genetic AS models, *I*_h_ amplitude is smaller in neocortical neurons and either larger or unchanged in thalamocortical (TC) neurons compared with nonepileptic strains. A lower expression of neocortical HCN subtype 1 channels is present in genetic AS-prone rats, and HCN subtype 2 knock-out mice exhibit ASs. Furthermore, whereas many studies have characterized *I*_h_ contribution to “absence-like” paroxysmal activity *in vitro*, no data are available on the specific role of cortical and thalamic HCN channels in behavioral seizures. Here, we show that the pharmacological block of HCN channels with the antagonist ZD7288 applied via reverse microdialysis in the ventrobasal thalamus (VB) of freely moving male Genetic Absence Epilepsy Rats from Strasbourg decreases TC neuron firing and abolishes spontaneous ASs. A similar effect is observed on γ-hydroxybutyric acid-elicited ASs in normal male Wistar rats. Moreover, thalamic knockdown of HCN channels via virally delivered shRNA into the VB of male Stargazer mice, another genetic AS model, decreases spontaneous ASs and *I*_h_-dependent electrophysiological properties of VB TC neurons. These findings provide the first evidence that block of TC neuron HCN channels prevents ASs and suggest that any potential anti-absence therapy that targets HCN channels should carefully consider the opposite role for cortical and thalamic *I*_h_ in the modulation of absence seizures.

**SIGNIFICANCE STATEMENT** Hyperpolarization-activated cyclic nucleotide-gated (HCN) channels play critical roles in the fine-tuning of cellular and network excitability and have been suggested to be a key element of the pathophysiological mechanism underlying absence seizures. However, the precise contribution of HCN channels in neocortical and thalamic neuronal populations to these nonconvulsive seizures is still controversial. In the present study, pharmacological block and genetic suppression of HCN channels in thalamocortical neurons in the ventrobasal thalamic nucleus leads to a marked reduction in absence seizures in one pharmacological and two genetic rodent models of absence seizures. These results provide the first evidence that block of TC neuron HCN channels prevents absence seizures.

## Introduction

Absence seizures (ASs), which consist of relatively brief periods of lack of consciousness accompanied by spike-and-wave discharges (SWDs) in the EEG, are a feature of many genetic generalized epilepsies and believed to be generated by abnormal neuronal activity in reciprocally connected neocortical and thalamic territories (Crunelli and Leresche, 2002; Blumenfeld, 2005). Among the different voltage-dependent channels that may be involved in the pathophysiological mechanisms of these nonconvulsive seizures, hyperpolarization-activated cyclic nucleotide-gated (HCN) channels, which are present in the vast majority of cortical and thalamic neurons, have been extensively investigated (Huang et al., 2009; Noam et al., 2011; Reid et al., 2012). However, the selective contribution of cortical versus thalamic HCN channels in ASs is still not fully understood (Noam et al., 2011).

Several studies in humans have reported an association between HCN channel mutations and genetic epilepsies: in particular, mutations in HCN subtype 1 (HCN1) and HCN subtype 2 (HCN2) were reported in patients with genetic generalized epilepsies (Tang et al., 2008; DiFrancesco et al., 2011), including febrile seizures and early infantile epileptic encephalopathy (Nakamura et al., 2013; Nava et al., 2014). However, it is difficult to draw any firm conclusion from these human studies since ASs are not the only phenotype present in these diverse forms of epilepsy.

As far as cellular effects are concerned, *in vitro* studies have shown that blocking the *I*_h_ current that HCN channels generate in thalamocortical (TC) neurons enhances bicuculline-elicited synchronized thalamic activity resembling absence paroxysms by increasing burst firing in TC neurons (Bal and McCormick, 1996). The observation that mice with spontaneous or induced genetic ablation of HCN2 channels exhibit ASs (Ludwig et al., 2003; Chung et al., 2009; Heuermann et al., 2016) has been interpreted as providing support to this view. However, since HCN2 channels are highly expressed in both cortical and thalamic neurons (Notomi and Shigemoto, 2004), these *in vivo* data cannot be used to draw any firm conclusion on a pro-absence role of thalamic HCN channels. Indeed, in genetic AS models, *I*_h_ of neocortical neurons is smaller (Strauss et al., 2004; Kole et al., 2007), resulting in increased temporal summation of EPSPs and enhanced burst firing (Strauss et al., 2004), whereas in TC neurons, *I*_h_ has been reported to be either larger or unchanged compared with nonepileptic strains (Kuisle et al., 2006; Kanyshkova et al., 2012; Cain et al., 2015) and the ability of burst firing is decreased (Cain et al., 2015). More importantly, the precise influence of HCN channels of thalamic versus cortical neurons on behavioral seizures has never been investigated. This, together with the complexity of the diverse cellular and synaptic effects that *I*_h_ can exert under normal conditions and their consequences on paroxysmal network excitability (Huang et al., 2009; Noam et al., 2011; Reid et al., 2012), makes it difficult to draw causal links between HCN channel function and ASs.

Here, we directly investigated the role of thalamic HCN channels in ASs using both pharmacological and genetic tools to selectively suppress HCN channel function in TC neurons in rodent models of absence epilepsy under freely moving conditions. We report that bilateral reverse microdialysis application of the HCN blocker ZD7288 into the ventrobasal thalamus (VB) blocks ASs in two well established absence models, the Genetic Absence Epilepsy Rats from Strasbourg (GAERS; Depaulis et al., 2015) and the γ-hydroxybutyric acid (GHB)-injected Wistar rats (Venzi et al., 2015), and decreases tonic, but not burst, firing in TC neurons of freely moving GAERS. Furthermore, silencing thalamic HCN gene expression with shRNA in the VB nucleus of Stargazer mice, another genetic absence epilepsy model (Fletcher and Frankel, 1999), is effective in reducing spontaneous ASs. Thus, in contrast to inferences from previous *in vitro* studies in thalamic slices (Kuisle et al., 2006; Kanyshkova et al., 2012) and *in vivo* investigations using brain-wide HCN channel manipulations (Ludwig et al., 2003; Chung et al., 2009), block of TC neuron HCN channels prevents ASs.

## Materials and Methods

All experimental procedures were performed in accordance with the UK Animals (Scientific Procedures) Act 1986 and local ethics committee and expert group guidelines (Lidster et al., 2015). All efforts were made to minimize animal suffering and the number of animals used. Experiments were performed on adult (2–5 months old) male Wistar (Harlan Laboratories) and GAERS (School of Bioscience, Cardiff University) rats and Stargazer mice (School of Bioscience, Cardiff University), which were maintained on a normal diet and under an 8.00 A.M. to 8.00 P.M. light-on/light-off regime.

### 

#### Surgical procedures for recordings under anesthesia

Implantation of microdialysis and silicone probes for recording under ketamine/xylazine anesthesia rats was performed as described by David et al. (2013) and Taylor et al. (2014). In brief, the initial dose of anesthetics (ketamine, 120 mg/kg; xylazine, 20 mg/kg) and the maintenance dose (ketamine, 42 mg · kg^−1^ · h^−1^; xylazine 7 mg · kg^−1^ · h^−1^) were injected intraperitoneally. Body temperature was maintained at 37°C with a heating pad and measured with a rectal probe. A microdialysis probe (CMA 12 Elite), with 2 mm dialysis membrane length, was slowly (500 μm every 5 min) inserted unilaterally into the VB thalamus (AP, −3.2 mm; ML, 5.3 mm; DV, −7 mm; Paxinos and Watson, 2007) at a 16° angle with respect to the vertical axis such that its final position would rest between 0.05 and 1 mm away from the tip of the silicone probe, which was subsequently inserted. Artificial CSF (ACSF) alone or containing ZD7288 (500 μm in the inlet tube) was then delivered through the dialysis at a constant flow rate of 1 μl per minute. A 32-channel silicone probe with four shanks (Buzsaki32L-CM32, NeuroNexus Technologies) was then slowly lowered into the VB (AP, −3.2 mm; ML, 2.8 mm; DV, −4.5 mm), and the full-band signal including unit activity was recorded during 40 min of ACSF and 1 h of ZD7288 reverse microdialysis injection.

#### Surgical procedures for EEG recordings in freely moving rats

Rats under isoflurane anesthesia were implanted bilaterally with guide cannulas for microdialysis probes so that their tips rested just above the VB (AP, −3.2 mm; ML, ±2.8 mm; DV, −4.5 mm). Frontal (AP, +2.0 mm; ML, ±2.0 mm) and parietal (AP, −1.8 mm; ML, ± 5.0 mm) EEG screws were then implanted, and the rats were allowed to recover for at least 5 d. Twenty-four hours before each experiment, microdialysis probes with 2 mm dialysis membrane were inserted into the VB guide cannulas. On the day of recording, the rat was connected to the recording apparatus to habituate to the recording cage for 1 h. While habituating, ACSF was delivered via the inlet tube of the dialysis probes at 1 μl/min to allow stabilization of the surrounding tissue. For GAERS, the recording session consisted of 1 h of ACSF injection followed by 100 min of administration of either ACSF or ZD7288 (1–500 μm in the inlet tube) solutions, while recording the EEG continuously throughout the recording session. For recording in GHB-injected rats, the 1 h habituation was followed by a 40 min period where either ACSF or ZD7288 (500 μm in the inlet tube) solutions were delivered through the inlet tubing. Then, either saline or γ-butyrolactone (GBL), a GHB pro-drug, was injected intraperitoneally (100 mg/kg), and the EEG was recorded for 1 h. Rats and mice were randomly assigned to receive either ACSF or ZD7288 first, followed by the other solution a week later. No animal was treated more than twice.

#### Neuronal recordings in freely moving rats

When microdialysis was combined with unit recordings in freely moving conditions, procedures similar to those described by Taylor et al. (2014) were used. First, one guide cannula was implanted with the silicone probe mounted on a microdrive and its tip placed above the VB. On the day of the experiment, the dialysis probe delivering ACSF was inserted into the guide cannula, and the microdrive was advanced until suitable thalamic units were found. A control period of 20 min was always allotted before delivering ZD7288 (500 μm in the inlet dialysis tube). Note that unless otherwise indicated, the concentration of ZD7288 is always expressed in the test and figures as that of the solution perfused in the dialysis inlet tube. The corresponding tissue concentration can be deduced considering the general dialysis recovery of 5–10% (Chan and Chan, 1999; David et al., 2013; Montandon and Horner, 2013).

#### HCN-targeting and nontargeting shRNA

The shRNA design is similar to that in our previously published papers (Chávez et al., 2014; Neuner et al., 2015). In brief, the HCN-targeting shRNA sequence (CAGGAGAAGTACAAGCAAGTAGA) was chosen to target a conserved region within the open reading frame of mouse and rat HCN1–4. A nontargeting shRNA (GAGGATCAAATTGATAGTAAACC), which showed no homology to any known genes, was used as a control. Both sequences were screened for sequence homology to other genes with NCBI-BLAST (www.ncbi.nlm.nih.gov/BLAST) and did not contain known immune response-inducing motifs (GTCCTTCAA, CTGAATT, TGTGT, GTTGTGT; Hornung et al., 2005; Judge et al., 2005; Robbins et al., 2009). In addition, both sequences follow rational designs developed for siRNAs (Amarzguioui and Prydz, 2004; Hsieh et al., 2004; Reynolds et al., 2004; Takasaki et al., 2004; Ui-Tei et al., 2004; Huesken et al., 2005; Vert et al., 2006; Ichihara et al., 2007; Katoh and Suzuki, 2007).

Desalted shRNA oligos containing a modified miR155 loop (GTTTTGGCCACTGACTGAC) and overhangs complementary to BamHI and XhoI restriction sites were custom synthesized (Invitrogen), resuspended using Duplex Buffer (Integrated DNA Technologies), and cloned into a “CreOff” adeno-associated virus (AAV) vector with a floxed cassette that contains a U6 polymerase III promoter to drive shRNA expression and a CMV promoter to drive eGFP expression for identification of transduced neurons. Constructs were cloned into pFB-AAV shuttle plasmids to allow for a baculovirus expression system-based AAV production. AAV constructs were maintained and propagated with Stbl3-competent cells (Invitrogen). Strict attention was paid to the integrity of the vector-inverted terminal repeats in plasmid preparations. All AAV plasmids were verified by diagnostic enzyme digestions. High-titer AAVs with serotype 9 were commercially produced by Virovek and included the green fluorescent protein eGFP under a CMV promoter (Chávez et al., 2014; Neuner et al., 2015) to label infected cells (see Fig. 7*A*).

#### Viral injection

Eighteen Stargazer mice were implanted with epidural fronto-parietal stainless steel EEG screws under isoflurane anesthesia, as described previously for rats. A craniotomy was performed above the VB (AP, −1.8 mm; ML, 1.5 mm; Paxinos and Watson, 2007), and an A10_l Gastight Hamilton syringe with a 34 GA needle that was filled with mineral oil and viral vector (see below) was inserted vertically. Needles were then lowered slowly into the thalamus (DV, −3.0 mm from the pia) and left in place for 10 min. The viral vector was diluted to a final titer of 2.18 × 10^13^ vg (viral genome copy)/ml (control, nontargeting, shRNA) and 1.145 × 10^13^ vg/ml (HCN-shRNA), injected bilaterally (2 × 500 nl) at a rate of 100 nl/min using a programmable micro-pump (UMP3–1; WPI), and allowed to disperse for an additional 10 min before the needle was slowly retracted.

Normal (3-month-old) male C57BL/6J mice were given injections of HCN-targeting (*n* = 6) and nontargeting (*n* = 7) shRNA (as described above) into the VB for investigating the effect of these shRNAs on the *in vitro* electrophysiological properties of TC neurons. Since the results from these normal mice were similar to those obtained from Stargazer mice, the electrophysiological data from the two strains were pooled.

#### Thalamic slice preparation, *in vitro* whole-cell recording, and data analysis

Thirty-two to thirty-six days after the viral injection, a modified method optimized for adult mice was used to prepare thalamic slices containing the VB (Ting et al., 2014). Briefly, mice were deeply anesthetized with ketamine/xylazine (80/8 mg/kg) and transcardially perfused with 20–25 ml of cold (4°C) ACSF containing (in mm) 93 *N*-methyl-d-glucamine, 2.5 KCl, 1.2 NaH_2_PO_4_, 30 NaHCO_3_, 20 HEPES, 25 glucose, 5 *N*-acetyl-l-cysteine, 5 Na-ascorbate, 3 Na-pyruvate, 10 MgSO_4_, and 0.5 CaCl_2_. The brains were then quickly removed from the skull, blocked, and sliced (320 μm thickness) in the coronal plane. After a short (12 min) recovery in a warmed (35°C) N-methyl-D-Glucamin ACSF, the slices were incubated at room temperature (20°C) in HEPES-holding ACSF containing (in mm) 30 NaCl, 2.5 KCl, 1.2 NaH_2_PO_4_, 1.3 NaHCO_3_, 20 HEPES, 25 glucose, 5 *N*-acetyl-l-cysteine, 5 Na-ascorbate, 3 Na-pyruvate, 3 CaCl_2_, and 1.5 MgSO_4_. For recording, slices were submerged in a chamber perfused with a warmed (35°C) continuously oxygenated (95% O_2_, 5% CO_2_) ACSF containing (in mm) 130 NaCl, 3.5 KCl, 1 KH_2_PO_4_, 24 NaHCO_3_, 1.5 MgSO_4_, 3 CaCl_2_, and 10 glucose.

Whole-cell patch-clamp recordings of TC neurons located in the VB were performed using an EPC9 amplifier (Heka Elektronik). Patch pipettes (tip resistance, 4–5 MΩ) were filled with an internal solution containing the following (in mm): 126 K-gluconate, 4 KCl, 4 ATP-Mg, 0.3 GTP-Na_2_, 10 HEPES, 10 kreatin-phosphate, and 8 biocytin, pH 7.25; osmolarity, 275 mOsm. The liquid junction potential (−13 mV) was corrected off-line. Access and series resistances were constantly monitored, and data from neurons with a >20% change from the initial value were discarded. The ratio of the input resistance at the peak (*R*_peak_) and that at the end of the 1-s-long voltage step (*R*_ss_; as illustrated in Fig. 5) was taken as a measurement of the depolarizing “sag” elicited by HCN channel activation. Action potential amplitude was measured from threshold (20 mV/ms on the first derivative of the membrane potential) to the peak of the action potential. Analysis of these whole-cell data was performed using custom routines written in Igor.

#### *In vivo* data acquisition and analysis

##### Spike sorting.

For unit recordings, signals were digitized with a 64-channel integrated recording system (version 2.3.0, 2006; Plexon) at 20 kHz with 16-bit resolution. EEG data were low-pass filtered with a windowed sinc filter at 100 Hz and downsampled to 200 Hz. Spike sorting and data preprocessing were performed with the Klusters, Neuroscope, NDManager, and Klustakwik software suites (Harris et al., 2000; Hazan et al., 2006). A typical high-frequency burst of action potentials of TC neurons was defined as a group of spikes that was separated by <7 ms, was preceded by a 100 ms period of electrical silence, and showed the characteristic decelerando pattern within a burst (Domich et al., 1986).

##### Spike-and-wave discharge analysis.

The EEG was recorded using an SBA4-v6 BioAmp amplifier (SuperTech), digitized at 1 kHz (Micro3 D.130; Cambridge Electronic Design) and analyzed with CED Spike2 version 7.3 and Matlab (R2011b; MathWorks). SWDs that accompanied behavioral ASs were detected semiautomatically with the aid of the SeizureDetect script (kindly provided by Dr. Steven Clifford, Cambridge Electronic Design) in Spike2 version 7.3 as described in detail by Venzi et al. (2016). For analysis of GAERS SWDs, data were normalized in two steps: first, all values were measured as percentage variation compared with the average values of the control periods, then all individual percentage values were recalculated as percentage change compared with the average value at each time point of the control group (set to 0% change). Only the second step of this calculation was applied to the SWDs of GHB and Stargazer data for which no control period exists. The time-frequency representation of SWDs was performed with a wavelet transform of SWD, as described by David et al. (2013). The frequency of SWDs was estimated from the distribution of the intervals that separate each spike-and-wave complex (SWC) extracted with the SeizureDetect program (Cambridge Electronic Design).

#### Histology

To examine the relative position of the tracks of the microdialysis and silicone probes, methods similar to those described by David et al. (2013) and Taylor et al. (2014) were used. Data were excluded from analysis if either the dialysis or the silicone probes were misplaced.

For HCN2 immunohistochemistry, brains were perfused with 4% PFA and stored in 0.1 m PB with 0.05% sodium azide at 4°C before slicing at 40 μm on a Vibratome (VT1000S; Leica Microsystems). After 1 h in 5% normal horse serum blocking solution, the sections were incubated in primary antibody rabbit anti-HCN2 (1:200; Alomone Labs) and diluted in 0.1 m Tris-buffered saline (pH 7.4)/0.1% Triton X-100 (Sigma) and 3% Normal Goat Serum, followed by secondary antibody Cy3 donkey anti-rabbit (1:500; Jackson ImmunoResearch Laboratories) and DAPI staining (1:200; Millipore), and mounted in Vectashield (Vector Laboratories) before imaging with a confocal microscope (FW 1000; Olympus). Quantitative analysis of HCN and GFP expression levels were performed with ImageJ software. Zones of interest of neuronal cell bodies were delimited manually, and the intensity was measured in the respective spectra (green, λ = 594 nm for HCN; red, λ = 488 nm for GFP). GFP green fluorescence intensity (in arbitrary units) was taken as an indicator of viral infection in a cell and correlated with the anti-HCN antibody red fluorescence intensity (see Fig. 6*D*).

#### Experimental design and statistical analysis

**Figure 1.**
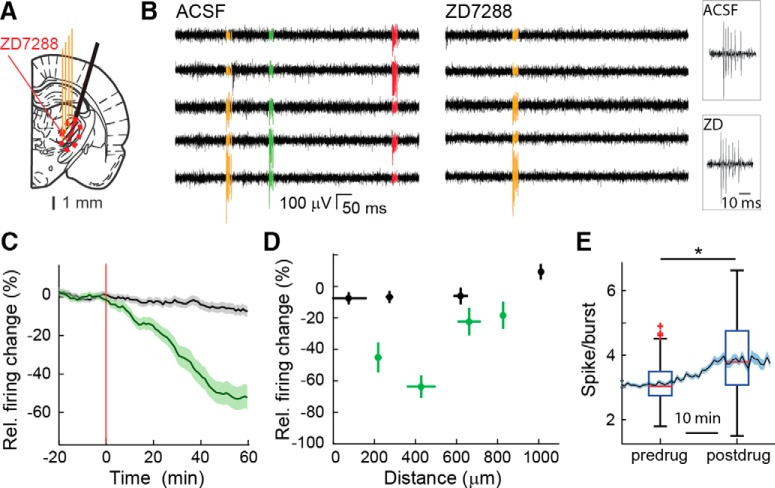
Temporal and spatial dynamics of the effect of ZD7288 applied by reverse microdialysis in the VB of ketamine/xylazine-anesthetized Wistar rats. ***A***, Position of the unilateral four-shank silicone probe (4 thin orange lines) and microdialysis probe (black thick oblique line) on a rat brain schematic drawing at the level of the VB [modified from Paxinos and Watson (2007)]. The red circled, striped area indicates the diffusion of ZD7288 as measured in ***D***. ***B***, Extracellular high-pass filtered traces from five adjacent contact points of a silicone probe show high-frequency bursts of action potentials of three clustered (color-coded) TC neurons during ACSF (left) and ZD7288 (right) microdialysis application. Right, Enlargement of bursts from the same TC neuron before (top) and during (bottom) ZD7288 (ZD) dialysis. The same y-scale is shown for all traces. ***C***, Time course of total firing of TC neurons during ACSF (black) and ZD7288 (green) microdialysis injection (500 μm in the inlet tube). Data are shown as percentage firing relative to that during ACSF (solid lines and shadows, mean ± SEM). The red vertical line (at time 0) indicates the start of ZD7288 application. Data from 87 and 45 neurons for ACSF and ZD7288, respectively, from 21 Wistar rats are shown (see Materials and Methods for further details). ***D***, Distance profile of the ZD7288 effect (green) on total firing compared with ACSF (black; same number of neurons as in ***C***). Horizontal bars indicate electrode position SDs relative to the dialysis membrane and calculated in 250 μm space bins, and vertical bars indicate SEM of ZD7288 effect. ***E***, ZD7288-elicited increase in the number of spikes per burst (*n* = 45 neurons; solid line and shadows, mean ± SEM). Time is centered on the half-time of the effect of ZD7288 estimated by a logistic function fit on the total firing rate variation after ZD7288 application. The box plot indicates median (red), upper and lower quartiles (box edges), extreme points (whiskers), and outliers (red crosses; see Material and Methods, Experimental design and statistical analysis). Median postdrug (3.75 spikes/burst) is significantly higher than predrug (3.02 spikes/burst; *p = 6.9 10^−5^, Wilcoxon signed rank test.).

**Figure 2.**
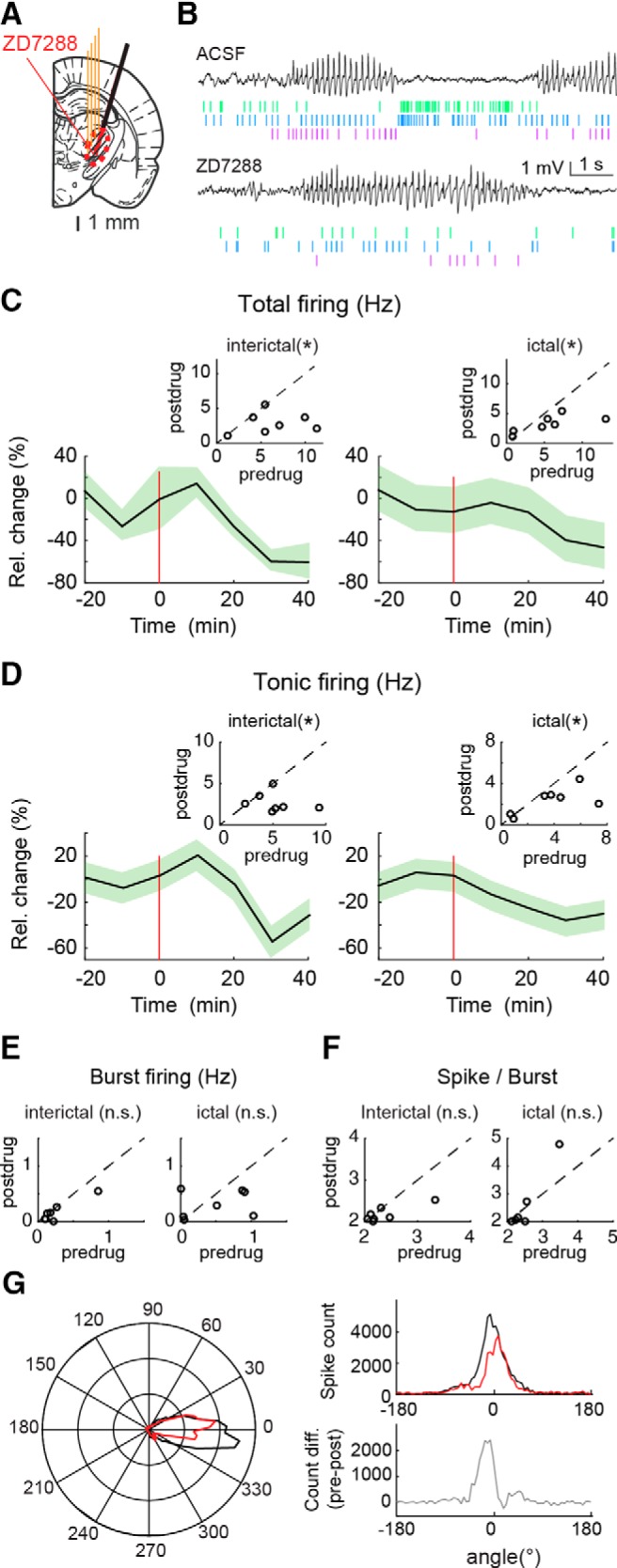
Effect of ZD7288 microdialysis injection in the VB on TC neuron firing in freely moving GAERS. ***A***, Position of the unilateral four-shank silicone probe (4 thin orange lines) and microdialysis probe (black thick oblique line) on a rat brain schematic drawing at the level of the VB [modified from Paxinos and Watson (2007)]. The red circled, striped area indicates the diffusion of ZD7288 as measured in Figure 1***D***. ***B***, Extracellular low-pass filtered traces from the silicone probe show ictal periods (with SWDs) and interictal periods, with below raster of clustered (color-coded) spikes of three TC neurons during ACSF (top traces) and ZD7288 (bottom traces) application. Note the drastic change of firing between ictal and interictal periods. ***C***, ***D***, Time course of total (***C***) and tonic (***D***) firing (solid line and shadows, mean ± SEM) during interictal periods (left) and during ASs (right) recorded during ACSF (data to the left of red horizontal line) and ZD7288 (data to the right of the red horizontal line) microdialysis application. Red vertical lines (at time 0) indicate the start of ZD7288 injection. The change in activity is illustrated by the inset plots that show total and tonic firing rate (hertz) for individual neurons during ACSF (predrug) versus ZD7288 (postdrug; with the black dashed line indicating equal predrug and postdrug values; *significant *p* values from left to right are 0.016, 0.039, 0.023, and 0.016, Wilcoxon signed rank test; *n* = 7 neurons). ***E***, ***F***, Plots, as inset plots in ***C*** and ***D***, showing the nonsignificant (n.s.) changes in burst firing and number of spikes per burst induced by ZD7288 microdialysis during interictal and ictal periods (*p* values from left to right are 0.078, 0.19, and 0.11, 0.5, Wilcoxon signed rank test; *n* = 7 neurons). ***G***, Left, Circular distribution plot of action potentials with respect to the SWC indicates a significant different distribution before (black line) and during (red line) ZD7288 application (*p* = 0.001, Kuiper 2-sample test; *n* = 58.8 10^3^ vs *n* = 40.6 10^3^ action potentials). Right, The maximal difference in the time distribution of action potentials between ACSF (black line, top plot) and ZD7288 (red line, top plot) occurs just before 0° (defined as the peak of the SWC), as highlighted by the subtraction of these two curves (gray line, bottom plot).

Experiments with reverse microdialysis on thalamocortical unit activity (Figs. 1, 2) were designed so that at least five neurons could be recorded per data point (David et al., 2013; McCafferty et al., 2018). Experiments involving SWD measurement involved a minimum of 6–11 animals, which in previous similar studies allowed statistical significance to be detected (Cope et al., 2009). Immunohistological procedures were performed on three animals per treatment group to collect enough thalamic slices (Cope et al., 2009).

Group comparisons were performed using the Wilcoxon signed rank test, and the Wilcoxon rank-sum test was used for paired or unpaired datasets. A logistic regression of the dose-dependent effect of ZD7288 on GAERS SWDs was performed with SigmaPlot. Linear regressions were performed for correlating the HCN-related fluorescence intensity to the GFP-related fluorescence intensity. Circular statistics was performed using the Kuiper two-sample test. All quantitative data in the text and figures are expressed as mean ± SEM. Values were defined as outliers if they were larger than *q*_3_ + *w*(*q*_3_ − *q*_1_) or smaller than *q*_1_ − *w*(*q*_3_ − *q*_1_), where *q*_1_ and *q*_3_ are the 25th and 75th percentiles, respectively, and *w* is 1.5, which corresponds to ±2.7 SDs for normally distributed data (as defined in Matlab; Mathworks).

## Results

### Time course and diffusion of microdialysis-applied ZD7288

We first characterized the time course and diffusion of the *I*_h_ antagonist ZD7288 applied via reverse microdialysis into the center of the VB, the thalamic nucleus somatotopic with the cortical “initiation site” of ASs in genetic rat models (Meeren et al., 2002; Polack et al., 2007). To this end, we measured the firing rate of TC neurons (the only neuronal population present in this thalamic nucleus) using a silicone probe closely positioned to a dialysis probe in ketamine/xylazine anesthetized Wistar rats (*n* = 21; Fig. 1*A*). Under this condition, the EEG mostly expressed sleep slow waves and TC neurons preferentially fired high-frequency bursts of action potentials (Fig. 1*B*). Unilateral application of 500 μm ZD7288 in the inlet dialysis tube, corresponding to a tissue concentration of 25–50 μm for a standard dialysis recovery of 5–10% (Chan and Chan, 1999; David et al., 2013; Montandon and Horner, 2013), led to a maximum and sustained firing reduction of ∼50% within 40 min from the start of the injection (Fig. 1*C*). This action was apparent in neurons located <600 μm from the dialysis probe but was absent in those located ≥600 μm away from the dialysis probe (Fig. 1*D*). As it has been previously reported in anesthetized rats during ZD7288 iontophoretic application (Budde et al., 2005), bursts recorded in the continuing presence of dialysis-applied ZD7288 were characterized by a significantly increased number of action potentials (*p* = 6.9.10^−5^, *n* = 45 neurons; Fig. 1*E*). Thus, in view of the dimensions of the rat VB thalamic nucleus (Paxinos and Watson, 2007), ZD7288 applied via a microdialysis probe placed in the middle of the VB is able to affect TC neuron firing in almost the entirety of this thalamic nucleus (Fig. 1*A*, red circled, striped area) and mostly sparing the nucleus reticularis thalamus (NRT), as we reported previously for a similarly applied Ca^2+^ channel blocker (David et al., 2013; Taylor et al., 2014).

### Neuronal effects of microdialysis-applied ZD7288 during ASs and interictal periods

No study so far has investigated the effect of *I*_h_ on TC neuron firing under natural conditions (i.e., in nonanesthetized animals), probably because of technical difficulties. Thus, having established the time course and diffusion of ZD7288, we then applied this antagonist by unilateral microdialysis into the VB while simultaneously recording firing activity of single TC neurons in a freely moving AS model, the GAERS (*n* = 3), with a close-by positioned silicone probe (Fig. 2*A*,*B*). In contrast with the increase observed in the same neuronal type *in vitro* (Lüthi et al., 1998), analysis of the activity of TC neurons (*n* = 7) showed that ZD7288 significantly decreased the total firing by ∼60 and 40% interictally and ictally, respectively (Fig. 2*C*). When different types of firing were analyzed individually, tonic firing was significantly reduced both ictally and interictally by ZD7288 (Fig. 2*D*), whereas burst firing was not (Fig. 2*E*). Importantly, in contrast to the results obtained under anesthesia (Fig. 1*E*), the number of spikes per burst in TC neurons recorded in freely moving rats was not significantly affected by ZD7288 (Fig. 2*F*). Finally, the time distribution of the extracellularly recorded action potentials with respect to the SWC (analyzed with circular statistics) was different between SWDs recorded during ACSF application and those during ZD7288 injection (ACSF mean angle, −2.2°; ZD7288 mean angle, 3.4°; *p* = 0.001, Kuiper test; Fig. 2*G*, left and top right plots), with the maximal difference between these two experimental conditions occurring just before 0° (Fig. 2*G*, bottom right plot).

### Pharmacological block of HCN channels in VB TC neurons impairs the expression of ASs

**Figure 3.**
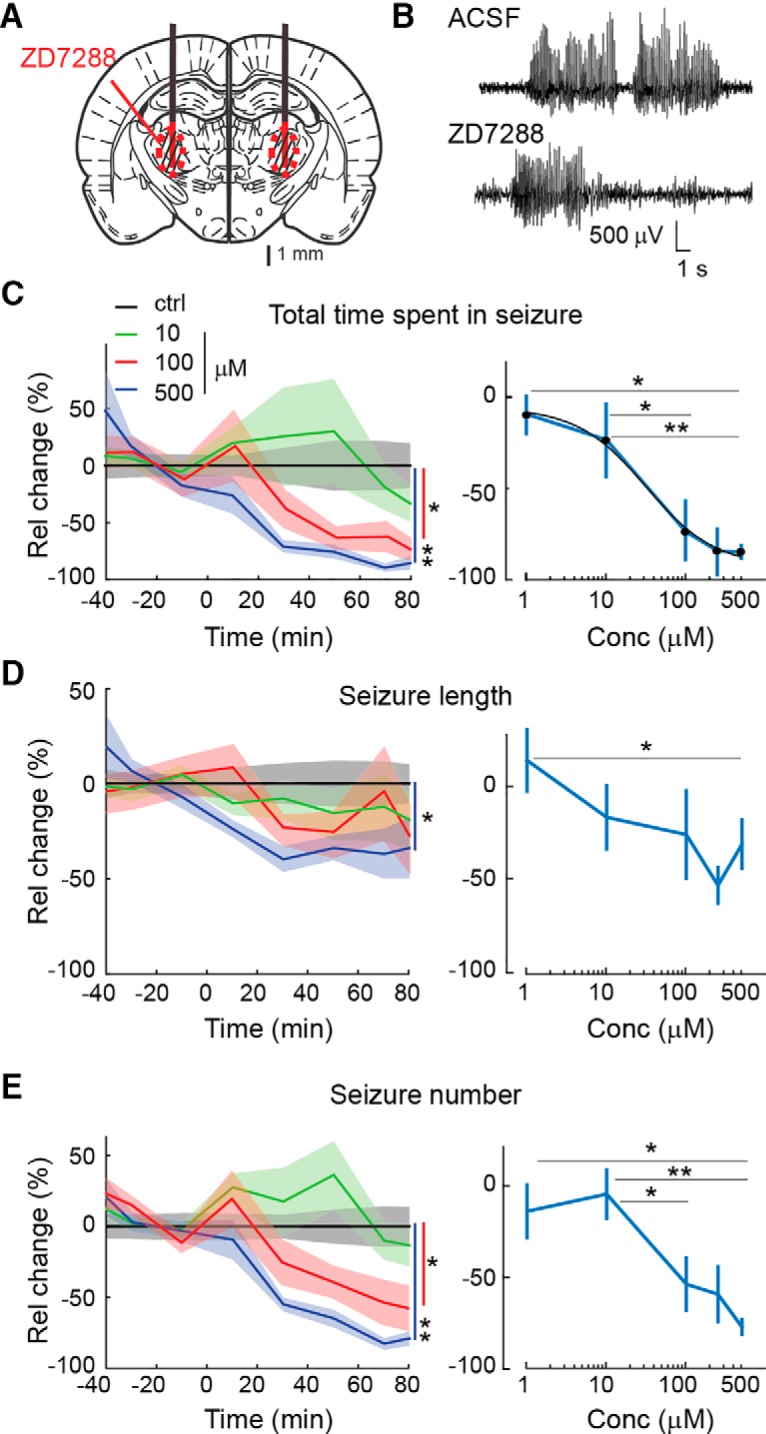
Effect of bilateral microdialysis injection of ZD7288 in the VB on ASs in freely moving GAERS. ***A***, Position of the bilateral microdialysis probes (black thick lines) and diffusion areas (red circled, striped areas) of ZD7288 are depicted on a rat brain schematic drawing at the level of the VB [modified from Paxinos and Watson (2007)]. ***B***, Representative EEG traces showing spontaneous SWDs during ACSF and ZD7288 (500 μm in the inlet tube) microdialysis application. ***C***, Time course (left) and concentration–response curve (right) of ZD7288 effect (solid line and shadows, mean ± SEM) on the total time spent in seizure normalized to ACSF values (see Materials and Methods for further details). The illustrated concentration color code refers to the ZD7288 concentration in the dialysis inlet tube. Time 0 indicates the start of ZD7288 dialysis. The number of animals is as follows: 9 (ACSF), 4 (1 μm), 8 (10 μm), 7 (100 μm), 2 (250 μm), and 6 (500 μm) [left, *p < 0.05, **p < 0.01, *p* = 0.023 (100 μm), and *p* = 4.10^−4^ (500 μm), Wilcoxon rank-sum test on averages between ACSF and 40–80 min data from the start of the ZD7288 application]. Absolute ACSF values (mean ± SEM) for the six reported time points are 295.4 ± 51.6, 308.8 ± 54.5, 276.7 ± 53.8, 275.2 ± 48.0, 219.1 ± 45.5, and 246.3 ± 52.5 s. A logistic fit of the concentration–response curve of ZD7288 indicate an EC_50_ of 29 μm. ***D***, Same as ***C*** for the length of individual seizures. Absolute ACSF values (mean ± SEM) are 7.91 ± 1.29, 7.64 ± 1.33, 8.24 ± 1.28, 7.96 ± 1.33, 7.29 ± 1.25, and 6.13 ± 0.83 s (left, *p = 0.025, Wilcoxon rank-sum test). ***E***, Same as ***C*** for the number of seizures. Absolute ACSF values (mean ± SEM) are 56.9 ± 10.4, 46.9 ± 8.6, 43.4 ± 9.1, 51.0 ± 11.7, 43.9 ± 11.2, and 45.6 ± 10.3 seizures (left, *p = 0.011, **p = 4.10^−4^, Wilcoxon rank-sum test).

We next investigated the effect of blocking *I*_h_ in VB TC neurons on spontaneous genetically determined ASs in freely moving GAERS (Fig. 3*A*). Application of ZD7288 by bilateral reverse microdialysis in the VB produced a marked and concentration-dependent (EC_50_ = 29 μm) decrease in the total time spent in seizures, with 500 μm almost abolishing ASs (82 ± 3%, *p* = 4.10^−4^, *n* = 6), whereas no significant effect was observed with 1 μm (*n* = 8; Fig. 3*B*,*C*). These effects were mostly driven by a marked reduction (75 ± 4%, *p* = 4.10^−4^) in the number of seizures (Fig. 3*E*), though a small decrease in the length of individual seizures (34 ± 13%, *p* = 0.025) was also observed (Fig. 3*D*).

**Figure 4.**
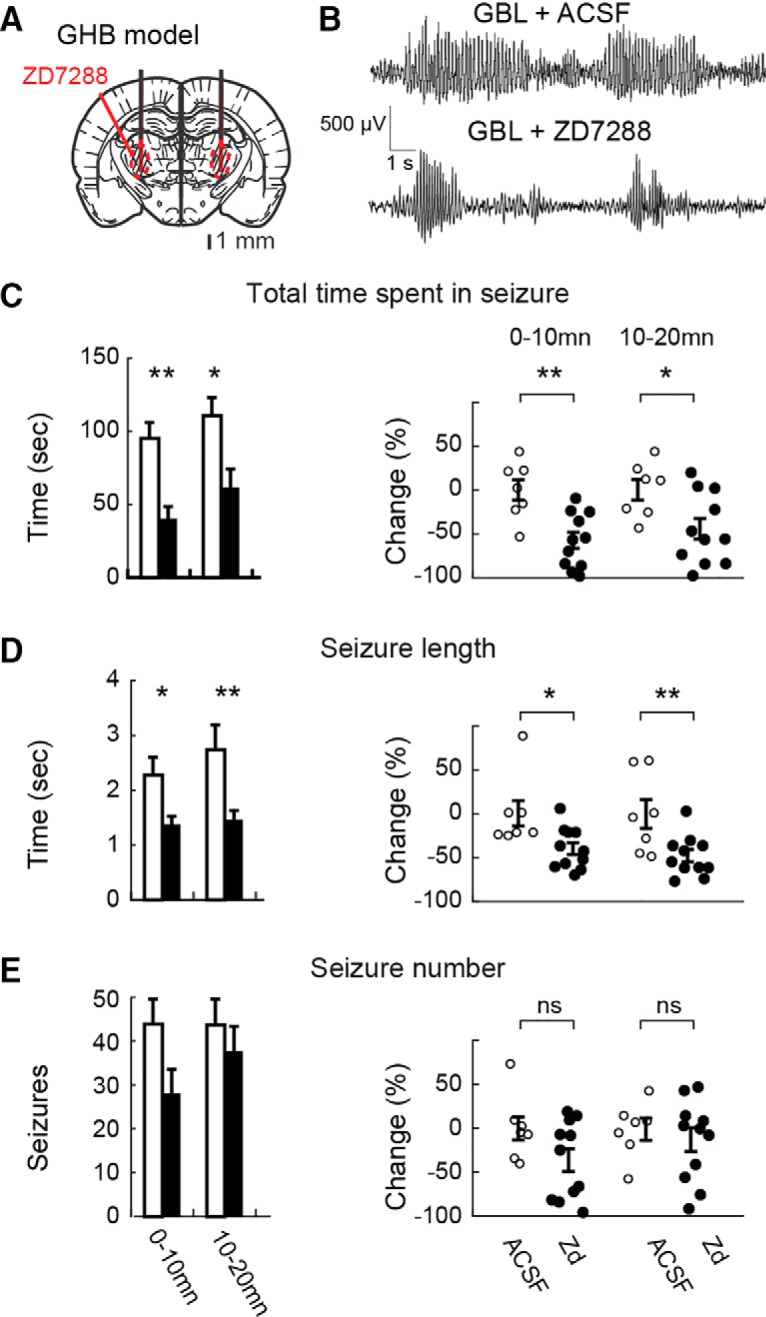
Effect of bilateral microdialysis administration of ZD7288 in the VB on GHB-elicited ASs in freely moving Wistar rats. ***A***, Position of the bilateral microdialysis probes (black thick lines) and diffusion areas of ZD7288 (red circled, striped area) are depicted on a rat brain schematic drawing at the level of the VB [modified from Paxinos and Watson (2007)]. ***B***, Representative EEG traces showing GHB-elicited SWDs during ACSF and ZD7288 (500 μm in the inlet tube) administration. ***C***, Left, Effect (mean ± SEM) of ZD7288 (500 μm, filled bars, *n* = 11 rats) versus ACSF (open bars, *n* = 7) on total time spent in seizures illustrated for 10 min bins. ZD7288 dialysis started 40 min before GHB injection (see Materials and Methods for further details). Right, Individual data points (open, ACSF; filled, ZD7288) for the 0–10 and 10–20 min time bins after GHB injection are normalized to data recorded during ACSF injection (**p = 9.4 10^4^, *p = 0.013, Wilcoxon rank-sum test, *n* = 7 and 11 animals in each group). ***D***, ***E***, Similar bar graphs (left) and scatter plots (right) as in ***C*** for average length of individual seizures (***D***; *p = 0.016, **p = 0.0082, Wilcoxon rank-sum test, *n* = 7 and 11 animals in each group) and number of seizures (***E***; *p* = 0.1, *p* = 0.34, Wilcoxon rank-sum test, *n* = 7 and 11 animals in each group).

Since genetically determined and pharmacologically induced ASs may depend on different cellular and network mechanisms (Crunelli and Leresche, 2002; Blumenfeld, 2005), the action of ZD7288 was then investigated in ASs elicited by systemic injection of a GHB prodrug, GBL (hereafter referred to as GHB; Venzi et al., 2015), in Wistar rats implanted with bilateral dialysis probes in the VB (Fig. 4*A*). Well separated ASs mainly occur up to 20–30 min after GBL administration (Fig. 4*B*; Venzi et al., 2015). Therefore, GHB was injected 40 min after the start of 500 μm ZD7288 microdialysis application, i.e., at a time when the effect of ZD7288 throughout the VB has reached steady state (compare Fig. 1*C*). As observed in GAERS, ZD7288 significantly decreased (58 ± 9%, *p* = 9.4.10^−4^, *n* = 11) the total time spent in seizures in the first 20 min after GHB injection (Fig. 4*C*). However, the ZD7288-elicited reduction was smaller than that observed in GAERS and was mainly attributable to a reduction in the length of individual seizures (40 ± 7%, *p* = 0.016; Fig. 4*D*) with no statistically significant effect on the number of seizures (Fig. 4*E*). No effect of ZD7288 on GHB-elicited ASs was observed beyond 20 min after GHB injection (data not shown). Thus, the pharmacological block of *I*_h_ in VB TC neurons by ZD7288 decreases both genetically determined and pharmacologically elicited ASs in freely moving animal models.

### Cellular effects of the HCN-targeting shRNA

**Figure 5.**
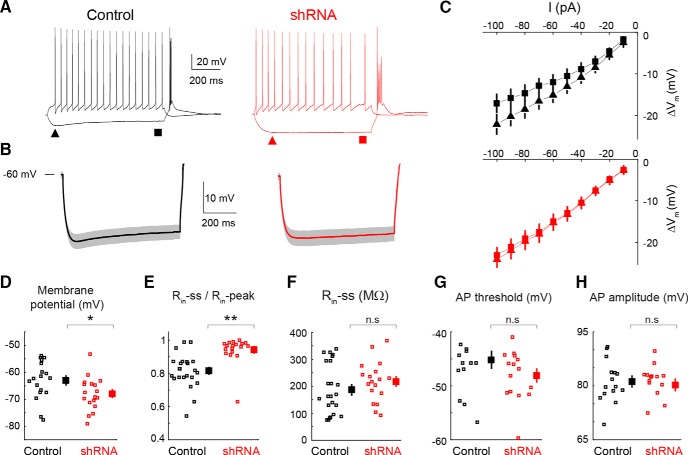
Effect of the HCN-targeting shRNA on the membrane properties of VB TC neurons *in vitro*. ***A***, Representative voltage responses of VB TC neurons to a hyperpolarizing and depolarizing current step (−100 and 50 pA, respectively) from nontargeting (black traces, control) and HCN-targeting shRNA-injected (red traces, shRNA) mice (membrane potential, −60 mV for both). Triangles and squares indicate the time of measurement of peak and steady-state input resistance (*R*_in_-peak and *R*_in_-ss, respectively, in the other panels). Note the lack of a depolarizing sag in the hyperpolarizing response of the HCN-targeting shRNA-injected neuron. ***B***, Averaged hyperpolarizing voltage responses (solid line, mean; shadow, ±SEM) in all recorded neurons show the marked reduction in the depolarizing sag in neurons injected with HCN-targeting shRNA (*n* = 18) compared with control (*n* = 18). ***C***, Voltage–current plots from all neurons show the lack of inward rectification in HCN-targeting shRNA-injected mice (triangles and squares indicate amplitude of hyperpolarizing pulse at peak and steady state, respectively, compare ***A***, ***B***). ***D–H***, Resting membrane potential (***D***), ratio of *R*_in_-ss and *R*_in_-peak (***E***), *R*_in_-ss (***F***), and action potential (AP) threshold (***G***) and amplitude (***H***) for neurons treated with nontargeting (black squares, control) and HCN-targeting shRNA (red squares, shRNA; large symbols indicate mean ± SEM; * and ** indicate statistical significance; n.s., not significant; *p* values are 0.032 (***D***), 6.2 10^−5^ (***E***), 0.56 (***F***), 0.17 (***G***), and 0.44 (***H***); Wilcoxon rank-sum test).

In addition to the pharmacological block, we investigated whether reducing the expression of HCN channels in the VB using shRNA could also suppress ASs. First, we assessed the functional effect of this genetic approach by monitoring the electrophysiological properties of VB TC neurons in slices taken from mice previously (32–36 d) given injections of either HCN-targeting or nontargeting shRNA in this thalamic nucleus (see Materials and Methods). Only TC neurons that showed eGFP fluorescence were patch clamped in slices from HCN-targeting shRNA mice. The resting membrane potential of TC neurons in slices from animals given injections of HCN shRNA (−68 ± 6 mV, *n* = 18) was more hyperpolarized than in mice that had received the nontargeting shRNA (−63 ± 7 mV, *n* = 18, *p* = 0.032; Fig. 5*D*). Moreover, the depolarizing sag of hyperpolarizing voltage steps was almost abolished in VB TC neurons infected with HCN-targeting shRNA compared with nontargeting shRNA (Fig. 5*A*,*B*), resulting in a similar input resistance at steady state (*R*_in_-ss) in the two groups (217 ± 75 MΩ, *n* = 18, and 186 ± 73 MΩ, *n* = 24, respectively; *p* = 0.56; Fig. 5*F*). Moreover, the steady-state and peak input resistance ratio (*R*_in_-ss/*R*_in_-peak) was significantly larger in neurons from HCN-targeting than nontargeting shRNA (0.94 ± 0.08 and 0.82 ± 0.01, *n* = 18 and 24, respectively; *p* = 6.2.10^−5^; Fig. 5*E*), indicating that the sag difference is not a consequence of a difference in *R*_in_. Application of ZD7288 (10 μm) to five TC neurons transfected with HCN-targeting shRNA abolished the small remaining sag (where present) but had no effect on the resting membrane potential (not shown). In contrast, action potential properties were not affected (threshold, −45 ± 6 vs −48 ± 5 mV; amplitude, 82 ± 2 mV vs 80 ± 2 mV; both *n* = 15 and *p* = 0.17 and *p* = 0.46, respectively; Fig. 5*G*,*H*). These data demonstrate that our HCN-targeting shRNA does selectively affect *I*_h_-dependent membrane properties of VB TC neurons without altering other neuronal properties.

### Genetic ablation of HCN channels reduces ASs

**Figure 6.**
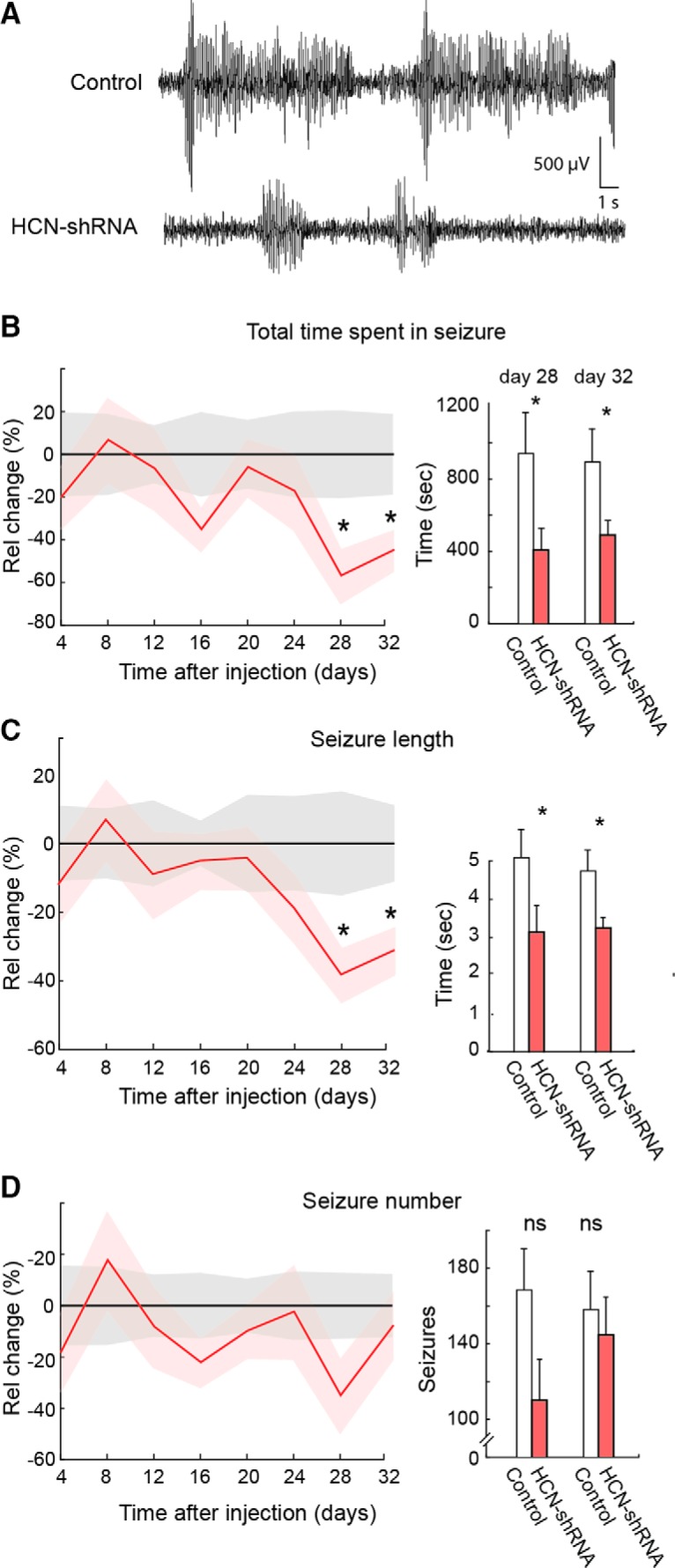
Effect of bilateral injection in the VB of an HCN shRNA on ASs in freely moving Stargazer mice. ***A***, Representative EEG traces showing spontaneous SWDs in a nontargeting control shRNA (top) and an HCN shRNA-injected Stargazer mouse (bottom). ***B–D***, Left, Effect of shRNA injection (solid line and shadows, mean ± SEM; red line, *n* = 9) on total time spent in seizure (***B***), length of individual seizures (***C***), and number of seizures (***D***) compared with nontargeting control shRNA (black line, *n* = 9) measured at the indicated days after shRNA injection (day 0). Values are normalized to control group mean (black line) for each time point. Right, Histograms of absolute values of total time, length of individual seizures, and number of seizures for test days 28 and 32 (3 h recordings; * indicates statistical significance; *p* values are 0.036 and 0.029 (***A***), 0.035and 0.043 (***B***), and 0.056 and 0.42 (***C***; Wilcoxon rank-sum test). Absolute values for the control group in all other test days were not different from those of days 28 and 32.

Having established the functional effect of the HCN-targeting shRNA on TC neuron membrane properties, we next assessed the effect of this genetic suppression of *I*_h_ on ASs in nine Stargazer mice, a monogenic mouse model of ASs (Fletcher and Frankel, 1999), which had received bilateral injection of viral construct into the VB. Another group (*n* = 9) of Stargazer mice received bilateral injections of a nontargeting shRNA. ASs were then monitored every 4 d for over 1 month. A statistically significant reduction in the total time spent in seizures (57 ± 12 and 45 ± 9%, *p* = 0.036 and *p* = 0.029, *n* = 9) and the average length of individual seizures (38 ± 7 and 31 ± 6%, both *p* = 0.035 and *p* = 0.043) was observed in HCN-targeting compared with nontargeting shRNA-injected mice at 28 and 32 d after injection, respectively (Fig. 6*A–C*). The reduction in the average number of seizures was not significant at both days (35 ± 14 and 8 ± 12%, respectively, *p* = 0.056 and *p* = 0.42; Fig. 6*D*).

**Figure 7.**
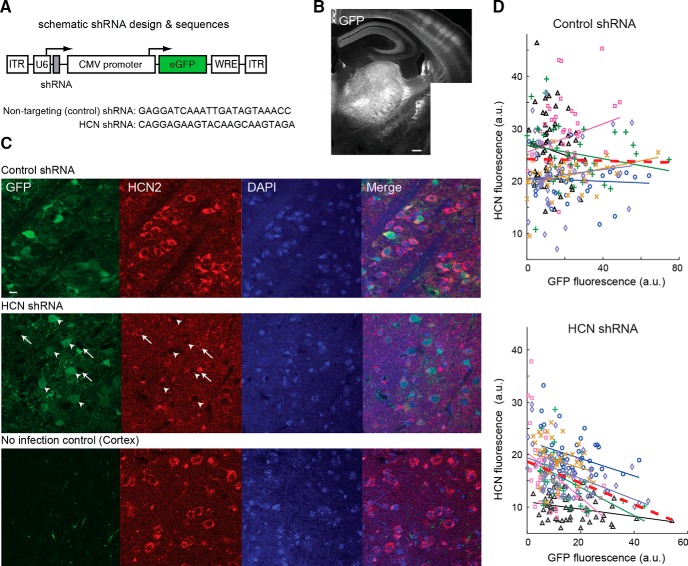
Genetic suppression of thalamic HCN channels decreases their expression in VB. ***A***, AAV construct includes eGFP and shRNA targeting HCN subunits or nontargeting HCN subunits. ***B***, Composite image showing GFP fluorescence restricted to thalamic nuclei and their projection to somatotopic cortical areas. Note barrels in somatosensory cortex. ***C***, Top row, Confocal images showing GFP and intrinsic HCN2 expression in DAPI-positive VB TC neurons from a Stargazer mouse that received injections of the nontargeting control shRNA. Middle row, Same for a mouse that received injections of the shRNA targeting the HCN sequence. Note the low level (or absence) of HCN fluorescence in those TC neurons that express a high level of GFP signal (white arrowheads) compared with cells expressing a low level of GFP (white arrows). Bottom row, Same for a cortical area of the same mouse. Note the absence of GFP-positive soma in the cortical section and the low level of HCN signal in the thalamic section (scale bar, 10 μm). ***D***, Quantifications of GFP and HCN expression in thalamic sections of Stargazer mice that received the nontargeting control shRNA (top) and the HCN targeting shRNA (bottom) for each neuron (symbols). Each line corresponds to the linear regression between the green (GFP) and the red (HCN) fluorescence of neurons from a single section. The same color line or symbols indicate cells of the same section. None of the correlations for the nontargeting control shRNA was significant (*p* = 0.31, 0.14, 0.23, 0.67, 0.051, and 0.59), whereas five of six sections from mice that received injections of the shRNA had a significant negative correlation (*p* = 0.041, 0.0006, 0.001, 0.03, 0.17, and 0.0001). Red dashed lines indicate the linear regression for the entire population of neurons (top, *p* = 0.77; bottom, *p* = 3.82 10^−9^).

At the end of the behavioral experiment (i.e., day 32 after injection), the brain of the Stargazer and wild-type mice, which had been injected, were harvested to measure GFP and HCN expression in thalamic and cortical slices (Fig. 7*B*). Triple labeling of VB TC neurons showed the colocalization of GFP, HCN2, and DAPI in all mice (Fig. 7*C*). As shown in Figure 7, *C* and *D*, in HCN shRNA-infected mice, TC neurons that were immunopositive for GFP had a low HCN immunoreactivity compared with nontargeting shRNA-infected animals. Indeed, a negative correlation was observed between HCN and GFP immunostaining in five of the six slices that had received the HCN shRNA (Fig. 7*D*, bottom), whereas no correlation was observed in all six mice that received injections of the missense RNA (Fig. 7*D*, top; linear regression *R*^2^ = 0.12, *p* = 3.82.10^−9^ vs *R*^2^ = 0.0004, *p* = 0.77 when pooling all data points together). Notably, the expression of the virus was restricted to the VB, as indicated by the data showing that (1) the GFP expression remained restricted to the thalamus and only projecting fibers were visible in the neocortex (Fig. 7*B*,*C*) and (2) cortical expression of HCN immunofluorescence was still prominent in the neocortex (Fig. 7*C*, bottom).

### Effect of thalamic *I*_h_ block on SWD parameters

**Figure 8.**
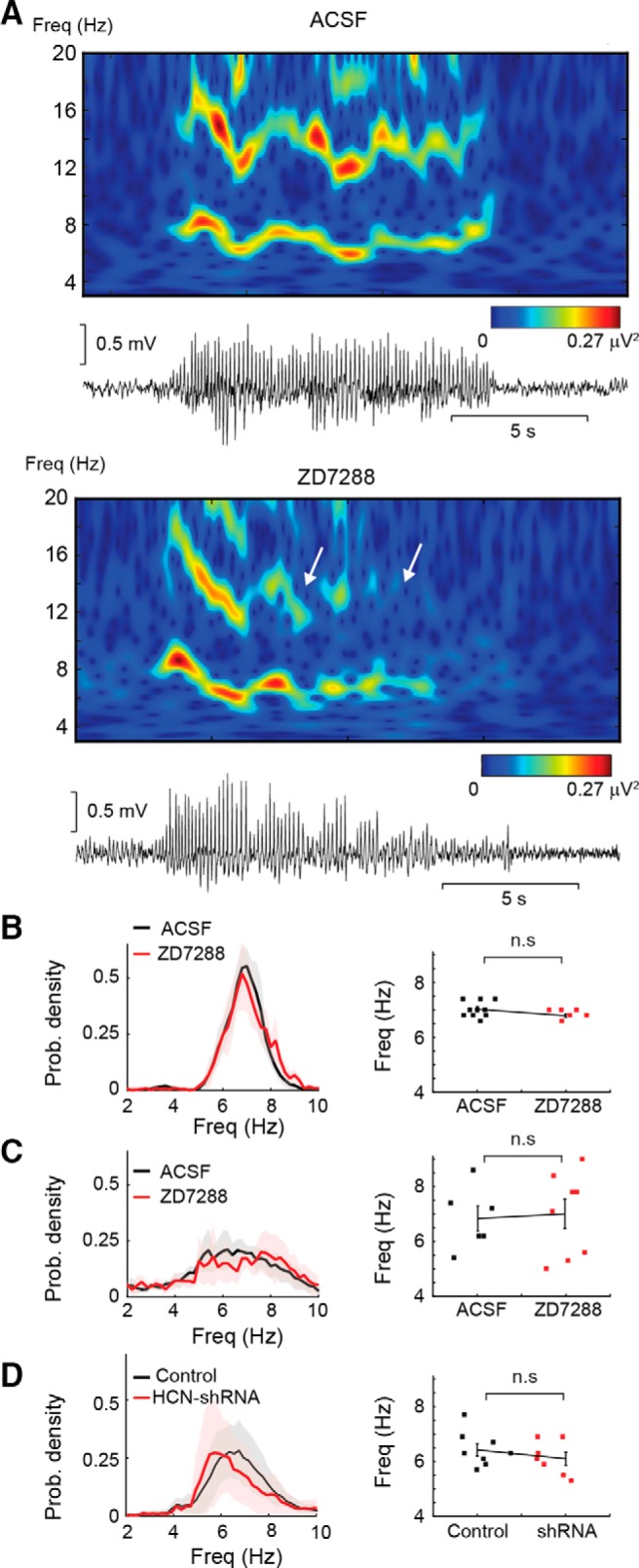
Effect of pharmacological and genetic suppression of *I*_h_ on SWD properties. ***A***, Representative examples of wavelet transform (top plots) of SWDs (bottom traces) before (ACSF) and during ZD7288 application. A clear loss of power is visible toward the end of the SWD recorded during ZD7288 application. A decrease in the first harmonic indicates a reduction of the spike component and an increase in the wave component of the SWD. ***B–D***, Frequency analysis of SWDs in the GAERS (***B***; *p* = 0.11, *n* = 9, *n* = 6) and GHB (***C***) models injected with ZD7288 (*p* = 0.83, *n* = 6, *n* = 8) and in Stargazer mice treated with HCN-targeting shRNA (***D***; *p* = 0.44, *n* = 8, *n* = 7; left, probability density plots of intervals between spike of SWCs; right, scatter plots of peak frequency for individual animals with mean ± SEM; Wilcoxon rank-sum test).

Finally, we compared some SWD parameters between control animals and those with a pharmacological or genetic suppression of thalamic HCN channel function. The time-frequency representation of SWDs indicated a decrease in the first harmonic (∼14 Hz) in the presence of ZD7288 in GAERS (Fig. 8*A*). To quantify this change, we calculated the averaged power spectra and found that the main frequency component of the SWDs at 7 Hz had a significantly increased power whereas the harmonic at ∼14 Hz was significantly smaller during the seizures that remained in the presence of ZD7288 in GAERS (Wilcoxon rank-sum test; control, *n* = 142; ZD7288, *n* = 45 seizures; *p* = 2.4.10^−6^; not shown). However, these changes were not observed after suppression of HCN channels with ZD7288 during GHB-elicited seizures and with shRNA in Stargazer mice. Moreover, the frequency of SWDs (estimated from the peak of interSWC-spike probability density; Fig. 8*B–D*, left) was not significantly different between control conditions and during the block of thalamic *I*_h_ for both spontaneous ASs in GAERS (control, 7.0 ± 0.1 Hz, *n* = 9; ZD7288, 6.8 ± 0.1 Hz, *n* = 6; *p* = 0.11) and in Stargazer mice (control, 6.4 ± 0.2 Hz, *n* = 7; shRNA, 6.1 ± 0.2 Hz, *n* = 8; *p* = 0.44; Fig. 8 *B*,*D*, right) as well as for GHB-elicited ASs (control, 6.8 ± 0.5 Hz, *n* = 6; ZD7288, 7.0 ± 0.5 Hz, *n* = 8; *p* = 0.82; Fig. 8*C*, right).

## Discussion

This study provides the first demonstration that (1) a reduction in *I*_h_ function in TC neurons of three animal models of absence epilepsy does reduce ASs and (2) the overall effect of blocking TC neuron HCN channels is a marked reduction in their firing rate both ictally and interictally. Therefore, in contrast to previous *in vitro* investigations and *in vivo* studies under anesthetic/neuroleptic regimes (Kuisle et al., 2006; Kanyshkova et al., 2012; Cain et al., 2015), these results demonstrate that *I*_h_ of TC neurons positively modulates the expression of ASs and support the view that the increased HCN channel function reported in TC neurons of genetic absence epilepsy models does contribute to and/or aggravate ASs and is not simply a seizure-related compensatory mechanism.

### Action of ZD7288 in freely moving animals

Before discussing the implications of our findings for ASs, it is important to consider some issues related to ZD7288 action. First, since ZD7288 concentration in the neuronal tissue is approximately one order of magnitude smaller than that in the inlet tube of the microdialysis probe (Chan and Chan, 1999), we are confident that the tissue concentrations achieved in our study are similar to those reported by us and others as selective for *I*_h_ (Harris and Constanti, 1995; Hughes et al., 1998; Blethyn et al., 2006). Indeed, we observed a significant effect on ASs at ZD7288 tissue concentrations as low as 10 μm. Moreover, the sigmoid shape of the ZD7288 concentration–response curve on GAERS ASs (Fig. 3*C*) speaks against an action on two different cellular targets under the freely moving conditions of this study. Indeed, in view of the standard 5–10% recovery rate of dialysis membranes, the EC_50_ (29 μm) of ZD7288 found here *in vivo* on the total time spent in seizures is similar to the 2 μm EC_50_ observed *in vitro* on *I*_h_ (Harris and Constanti, 1995). It is also unlikely that ZD7288 effect on ASs is mediated by an unselective action on Na^+^ channels since under the same microdialysis conditions ZD7288 decreases tonic, but not burst, firing of TC neurons in freely moving GAERS. Finally, the similarity in the effect on ASs with either the shRNA-elicited or ZD7288-mediated reduction of HCN channels in Stargazer or GAERS and GHB models, respectively, indicates that ZD7288 action under our experimental freely moving conditions is selective for *I*_h_.

Second, the ability of ZD7288 to affect GHB-elicited ASs only in the first 20 min after GHB administration should not be surprising since we recently showed that it is only in this initial period after injection that GHB elicits well separated bona fide ASs (with their clear behavioral and EEG components), whereas subsequent activity is characterized by a behavior more consistent with sedation/hypnosis and is accompanied by continuous low-frequency waves in the EEG (Venzi et al., 2015).

Third, a presynaptic, non-*I*_h_-mediated action of ZD7288, which is present at concentrations known to affect *I*_h_, was reported at hippocampal synapses (Chevaleyre and Castillo, 2002; Mellor et al., 2002). However, this ZD7288 effect is absent at neuromuscular junctions (Beaumont and Zucker, 2000; Beaumont et al., 2002) and has not been investigated at TC neuron synapses. Moreover, all the above data were obtained *in vitro*, and thus it is not known whether this presynaptic, non-*I*_h_-mediated action of ZD7288 occurs *in vivo* in freely moving animals (as those used in the present study), a condition where because of the more depolarized membrane potential than in *in vitro* experiments the voltage-dependent K^+^ current(s) that might underlie this ZD7288 effect (Chevaleyre and Castillo, 2002) may not be operative. Indeed, the similarity of the action of ZD7288 and the HCN-targeting shRNA support the view that the observed effect of ZD7288 on genetically determined and pharmacologically induced ASs occur via this drug action on *I*_h_ of TC neurons.

### *I*_h_ modulation of TC neuron ictal firing

Microdialysis application of ZD7288 in the GAERS VB increased the burst duration in TC neurons during ketamine/xylazine anesthesia, as shown previously in WAG/Rij rats under pentobarbital or neuroleptic regime (Budde et al., 2005). In contrast, in freely moving GAERS, ZD7288 did not affect interictal and ictal burst firing and burst duration, whereas total and tonic firing were decreased both between and during ASs. This differential action of ZD7288 on the two patterns of TC neuron firing is intriguing: it may be that the removal of the depolarizing influence of *I*_h_ has little effect on burst firing as TC neurons are relatively depolarized during ASs (Pinault et al., 1998), whereas it easily affects tonic firing. Alternatively, the somatodendritic distribution of HCN channel subtypes in TC neurons (Abbas et al., 2006) may contribute differently to the generation of tonic and burst firing (Connelly et al., 2015, 2016). Finally, the increase in tonic GABA_A_ current that is present in TC neurons of the GAERS, Stargazer, and GHB models (Cope et al., 2009) may differently offset the action of a decreased *I*_h_ on the summation of ictal corticothalamic EPSPs in these neurons (Ying et al., 2007), as it has been shown in cortical pyramidal neurons (Chen et al., 2010).

The recent characterization of the firing dynamics of thalamic neurons in freely moving GAERS and GHB models show that during ASs single TC neurons are mostly electrically silent or fire single action potentials, with T-type Ca^2+^ channel-mediated bursts of action potentials occurring rarely (McCafferty et al., 2018). Moreover, block of T-type Ca^2+^ channels of TC neurons does not affect behavioral ASs and the synchrony of the ictal thalamic output to the neocortex (McCafferty et al., 2018). These data, together with (1) the ZD7288-induced reduction of tonic but not burst firing (Fig. 2) and (2) the block of behavioral ASs after the pharmacological or genetic suppression of TC neuron HCN channels (Figs. 3, 4, 6), suggest that the most likely role for HCN channels of TC neurons in ASs is a contribution to the membrane potential: thus, the block of HCN channels of TC neurons will hyperpolarize these neurons, decreasing the synchronized thalamic output to the neocortex, thus compromising the re-engagement of the cortical network during ongoing seizures and ultimately being responsible for the reduction of ASs. Importantly, although the hyperpolarization induced by the block of *I*_h_ may increase T-type Ca^2+^ channel availability and thus the generation of a low-threshold spike, as observed in thalamic slices and in the whole animal under an anesthesia/neurolept regimen (Fig. 1; Budde et al., 2005), burst firing itself does not increase during ictal activity in the presence of ZD7288 in freely moving animals (Fig. 2), probably because of the less negative membrane potential in the latter than in the former vigilance state.

### Opposite role for cortical and thalamic *I*_h_ in ASs

In the WAG/Rij and GAERS models, different, and at times contrasting, results have been reported on *I*_h_ of TC neurons (in either VB or dorsolateral geniculate nucleus), including a clear increase in amplitude (Cain et al., 2015), responsible for the reduced burst firing, an increased channel density but a hyperpolarized *V*_[1/2]_ (Kanyshkova et al., 2012), or no apparent alteration in amplitude but an altered response to cAMP (Kuisle et al., 2006). The increased *I*_h_ of GAERS TC neurons has been suggested to be responsible for the reduced burst firing *in vitro* (Cain et al., 2015). In contrast, spontaneous or induced ablation of HCN2 channels leads to ASs and an enhanced ability to generate burst firing in TC neurons *in vitro* (Ludwig et al., 2003; Chung et al., 2009; Heuermann et al., 2016).

Our present results provide direct evidence that a pharmacological or genetic block of HCN channels in TC neurons reduces behavioral ASs in three freely moving absence epilepsy models. Although all these data may appear controversial, their apparent disagreement may originate from the “thalamocentric” interpretation of *in vivo* data obtained from brain-wide genetic manipulations that had explained these results on ASs by almost exclusive effects on thalamic network activity discarding any contribution by cortical HCN channels. Thus, in view of our results, it is more likely that the pro-absence effect of global HCN2 knock-out in normal mice (Ludwig et al., 2003) results from a cortical *I*_h_ loss of function. Similarly, a developmental decrease of HCN1 (but not HCN2) channels that leads to an *I*_h_ loss of function in the apical dendrites of layers 5 pyramidal neurons has been reported in the WAG/Rij absence model (Kole et al., 2007). In contrast, global HCN1 knock-out mice do not show an absence phenotype (Chen et al., 2009; Zhou et al., 2013), and *I*_h_ is increased in the soma of GAERS cortical layer 5/6 neurons (Williams et al., 2016). Whether these contradictory cortical data stems from compensatory changes in knock-out mice or are simply a reflection of opposite changes in cortical *I*_h_ in diverse models (Di Pasquale et al., 1997; Strauss et al., 2004) remains to be investigated.

In conclusion, using a pharmacological and a genetic approach to selectively suppress HCN channel function in TC neurons of three well established AS models, this study provides conclusive evidence on the longstanding controversial role for thalamic *I*_h_ in ASs by demonstrating that block of HCN channels of TC neurons prevents absence seizures.
